# Knowing the intention behind limb movements of a partner increases embodiment towards the limb of joint avatar

**DOI:** 10.1038/s41598-022-15932-x

**Published:** 2022-07-26

**Authors:** Harin Hapuarachchi, Michiteru Kitazaki

**Affiliations:** Department of Computer Science and Engineering, Toyohashi University of Technology, 1-1 Hibarigaoka, Toyohashi, Aichi 4418580 Japan

**Keywords:** Human behaviour, Motor control

## Abstract

We explored a concept called “virtual co-embodiment”, which enables users to share their virtual avatars with others. Co-embodiment of avatars and robots can be applied for collaboratively performing complicated tasks, skill training, rehabilitation, and aiding disabled users. We conducted an experiment where two users could co-embody one “joint avatar” in first person view and control different arms to collaboratively perform three types of reaching tasks. We measured their senses of agency and ownership towards the two arms of the avatar and changes in skin conductance levels in response to visual stimuli threatening the two virtual arms. We found that sense of agency, ownership, and skin conductance were significantly higher towards the virtual arm with control compared to the arm controlled by the partner. Furthermore, the senses of agency and ownership towards the arm controlled by the partner were significantly higher when the participant dyads shared a common intention or when they were allowed to see their partner’s target, compared to when the partner’s target was invisible. These results show that while embodiment towards partner-controlled limbs is lower compared to limbs with control, visual information necessary for predicting the partner’s intentions can significantly enhance embodiment towards partner-controlled limbs during virtual co-embodiment.

## Introduction

In recent years, with the development of Virtual Reality (VR) and Augmented Reality (AR) devices and software, social VR platforms and cyberspace have become easily accessible to users from various backgrounds. This has allowed more users to engage in activities such as socializing, gaming, participating in meetings, working, teaching, skill learning etc. virtually. Tracking and reflecting body movements into virtual avatars in real time has made “embodying” avatars possible with an enhanced “sense of embodiment”^1^ where users can experience and take full control over avatars in first person view to perform various tasks in virtual environments, inducing “sense of body ownership”^2,3^ and “sense of agency”^4,5^ towards their virtual avatars. A single user embodying one humanoid avatar to control all of its limbs as he/she would control his/her own body in real world is the most conventional way of avatar embodiment^1,6^. In this study, we explore a new concept called “virtual co-embodiment” where two or more users embody one avatar to collaboratively perform joint tasks. Co-embodiment can be achieved in several ways such as reflecting average movements (or movements in different ratios of control) of multiple users to one avatar/robot^7,8^, allowing multiple users to control different sections of one avatar limb/robotic arm^9^, and allowing each user to fully control separate limbs of one avatar^10,11^. We are focusing on this third method; We utilized an avatar of which left and right limbs could be controlled by two different individuals at the same time while both of them were immersed in the first-person view of the virtual avatar. We used this avatar to investigate how humans feel sense of embodiment towards limbs of their bodies controlled completely by others. A previous study with a special setting similar to “Ninin Baori” in a real environment (Participants viewed themselves in a mirror while another person behind them put hands forward as if they were the participants’ hands) on experiencing control over the movements of others suggests that when participants could hear instructions previewing movement of limbs controlled by another, they reported an enhanced feeling of controlling the hands that belonged to another person^12^. In this study, we used a new paradigm to further explore the effect of knowing the intentions behind arm movements on sense of embodiment towards an arm controlled by a partner during virtual co-embodiment.

**Figure 1:**
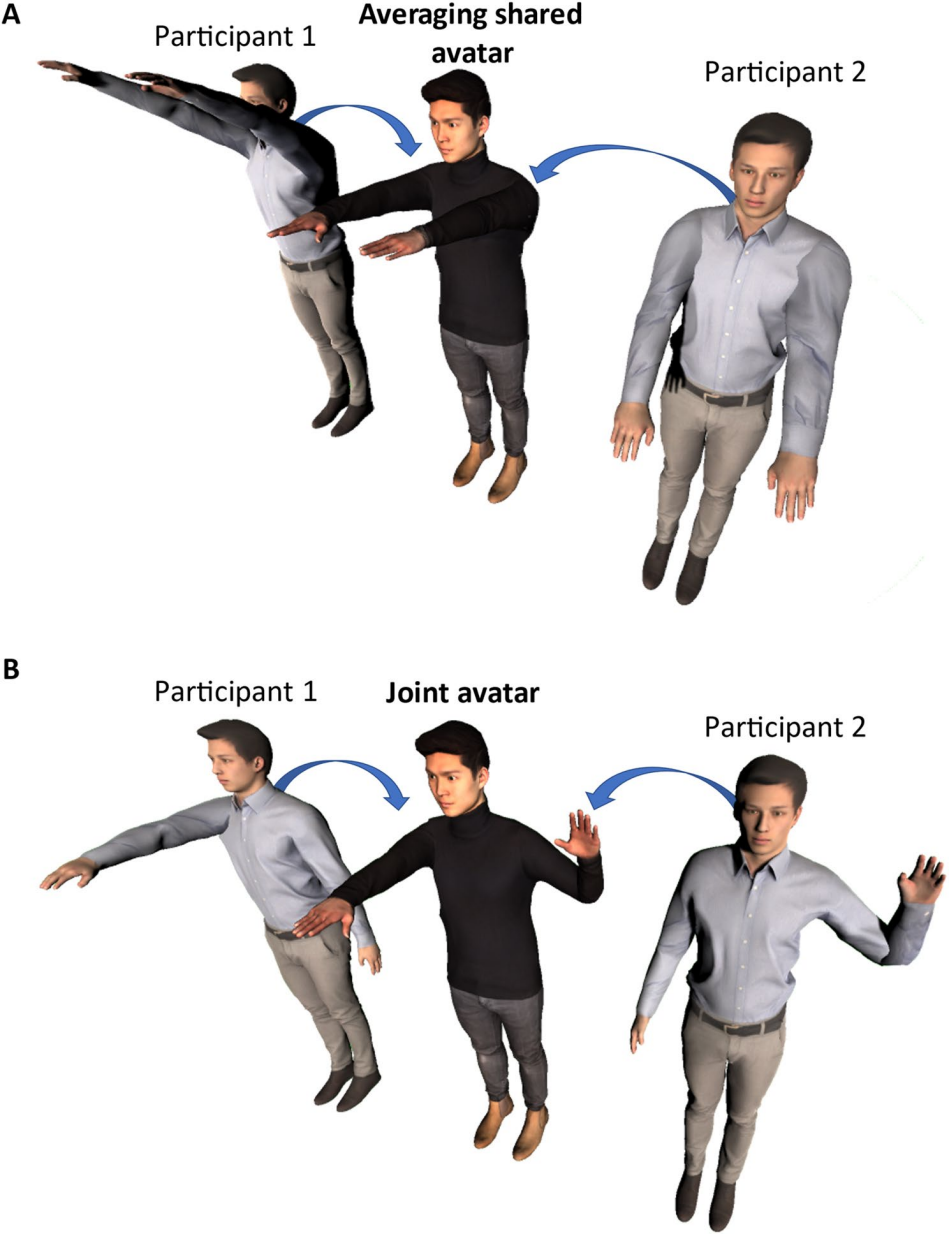
Two types of co-embodiment avatar: (**A**) A shared body avatar is controlled by two individuals, and their movements are averaged and reflected in the shared avatar^7,8^. (**B**) A joint body avatar is an avatar in which different body parts such as left and right half bodies are controlled by two different individuals.

Although the concept of two individuals co-embodying one avatar in first-person view is rather new, it has been proposed in a few recent studies (Fig. 1A). Hagiwara et al.^8^ conducted an experiment where two human participants were embodied within a concurrent shared avatar (Fig. 1A) in VR and showed that the movements of the shared avatar were straighter, and less jerky compared to the movements of the individual participants and the solo body avatar. Fribourg et al. ^7^ introduced the concept of “virtual co-embodiment” and showed that the participants were good at estimating their real levels of control but significantly overestimated their “sense of agency” when they could anticipate the motion of the avatar. These previous studies on virtual co-embodiment have studied avatars of which the movements were determined by averaging the movements of the two participants in real time (occasionally in different ratios of control).

Although averaging movements may have its benefits for some tasks for normal VR users, allowing a partner to completely control the virtual avatar’s limb corresponding to a person’s missing/immovable limb would be more applicable in the case of disabled VR users such as hemiplegic patients and amputees. Such aid by partners would allow amputees to weaken disabilities and perform complicated tasks in one avatar where each participant can focus on one detailed sub task. Hagiwara et al. ^9^ developed a robotic arm application where two users can control different sections (ie., elbow and wrist) of a robot arm to perform complicated tasks with more accuracy where the users can divide work and focus on one detailed sub task to collaboratively increase task performance of the overall task. Inspired by such studies, we have developed a new type of co-embodiment avatar called a “joint avatar”, in which the left half of the avatar is controlled by a user and the right half of the avatar is controlled by another (Fig. 1B). However, allowing another person to fully control a limb of a virtual avatar in which an individual is immersed in first person perspective may cause a severe lack of “sense of embodiment” towards that limb, which may lead to an uncomfortable experience associated with the feeling of being partly possessed by someone else. Therefore, addressing this lack of sense of embodiment towards limbs controlled by others is necessary for a pleasant virtual co-embodiment.

In this study, using this joint avatar, we investigated whether sense of embodiment towards a limb fully controlled by a partner would change depending on the nature of the task itself performed in the co-embodied/joint avatar. The task performed in the joint avatar can be considered as a 'joint action'. Sebanz et al.^13^ defined joint action as any form of social interaction whereby two or more individuals “coordinate” their actions in space and time to bring about a change in the environment. For example, two people carrying a table together would require both of them to coordinate their actions in a temporally and spatially precise manner to successfully execute the task. Some coordination mechanisms depend on sensorimotor information shared between co-actors, thereby making joint attention, prediction, non-verbal communication, or the sharing of emotional states possible^14^. Previous studies related to joint attention have suggested that the ability to direct one’s attention to where an interaction partner is attending provides a basic mechanism for sharing representations of objects and events^15,16^ which may possibly encourage the ‘we’ intention or the ‘we-mode’^17^ believed by some philosophers to be what joint action mostly depends on^16^. Here, we thought of the possibility of enhancing embodiment towards a co-embodied avatar by encouraging we-mode through sensory (visual) information manipulation during joint action, to eventually investigate on the relationship between embodiment and joint attention (or the sensory information responsible for joint attention).

Therefore, we hypothesized there to be an increase in embodiment if the individual and the partner shared one common intention/goal compared to having their own separate intentions before observing the partner’s action during the task (H1). Furthermore, we hypothesized that in the case of having two different targets, the embodiment would be higher when the partner’s target is visible compared to when it is invisible (H2).

### H1

Embodiment towards the limb controlled by a partner is higher when the goal is common to the partner.

### H2

Embodiment towards the limb controlled by a partner is higher when the partner’s goal is visible.

**Figure 2:**
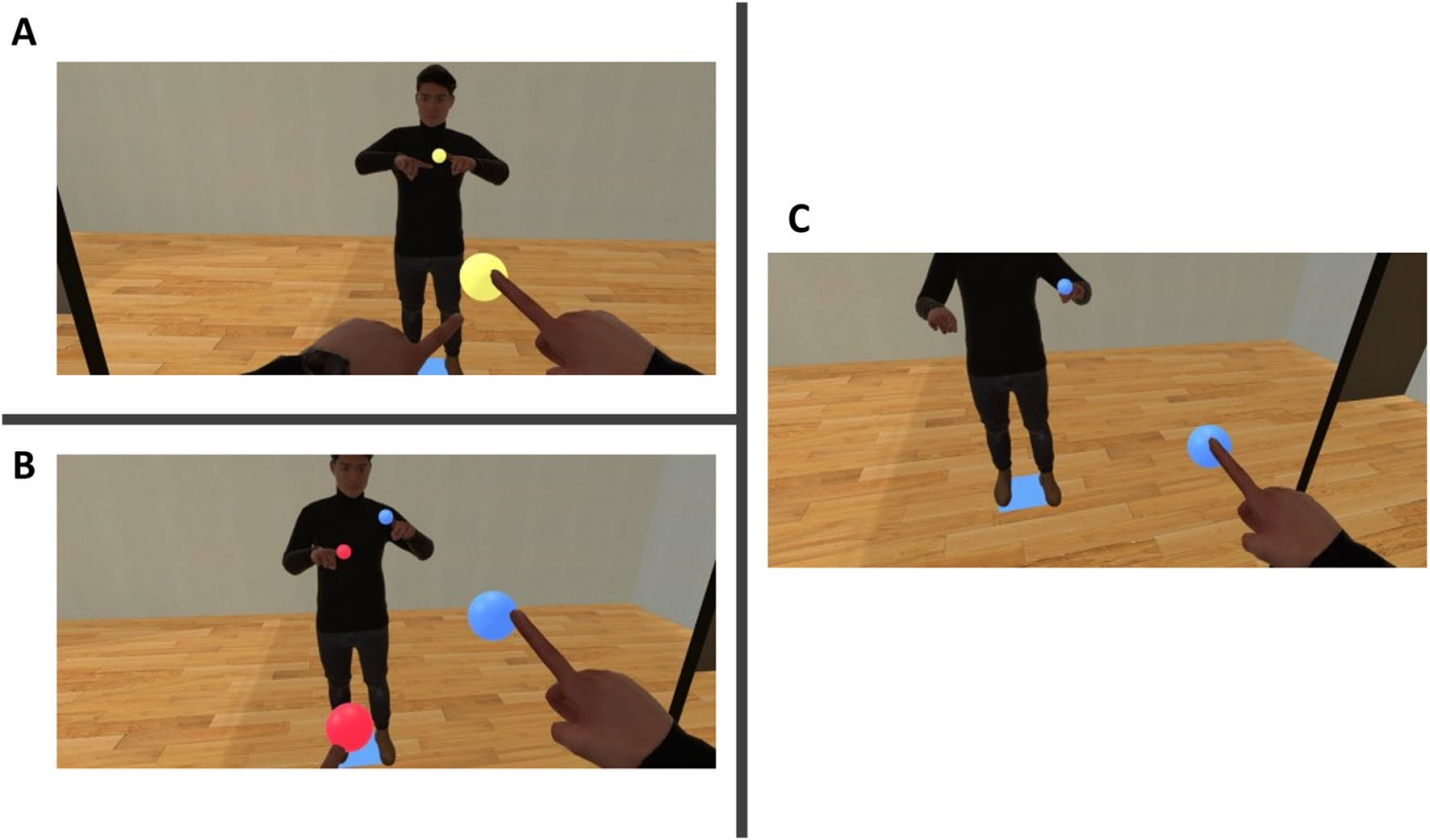
Experiment conditions: (**A**) *Same goal* condition, where the participants reached one yellow target with both hands of the joint avatar. (**B**) *Different goals: Visible* condition, where the participants reached red targets with the left hand and blue targets with the right hand of the joint avatar. (**C**) *Different goals: Invisible* condition, where the participants reached red targets with the left hand and blue targets with the right hand of the joint avatar but were only able to see the targets that could be reached with the hand they could control. Here, only blue targets were visible to the participant controlling the right arm while only red targets were visible to the participant controlling the left arm (image shows the view of the participant controlling the right arm).

To test these hypotheses, we conducted an experiment using the joint avatar. Participant dyads performed three types of reaching tasks involving and not involving joint action (Fig. 2).

**Figure 3:**
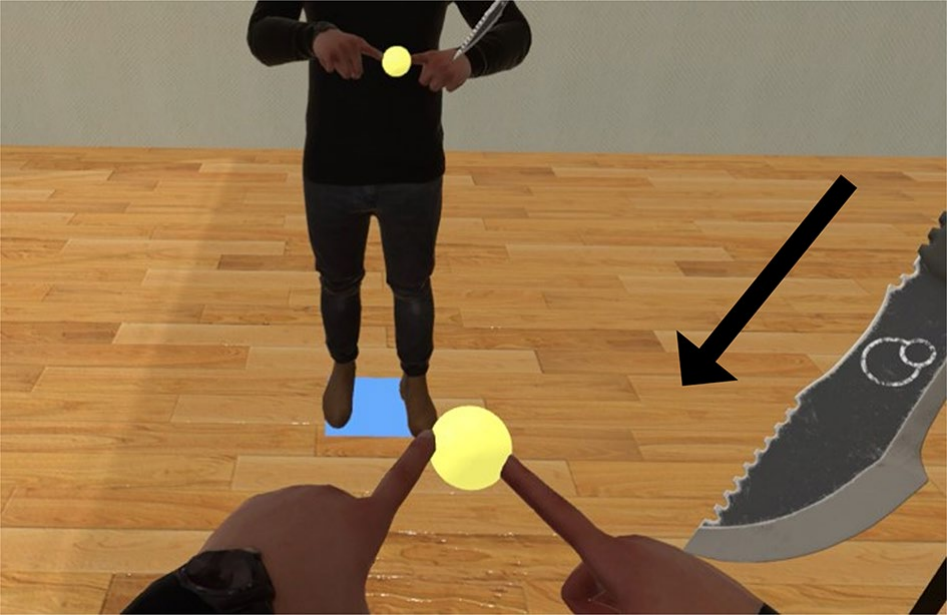
Stimulus of the knife at the end of the reaching task: example scene of the joint avatar’s right hand being stabbed at the end of the *Same goal* condition.

Multiple previous studies regarding ownership illusions towards rubber hands^18–20^ have shown that the increase in skin conductance level in response to threatening stimuli is higher towards objects with ownership illusions. For example, skin conductance when a strong blow with a hammer was given to a fake hand after tactile stimulation that induces an ownership illusion towards the rubber hand, the participants have shown a higher increase in skin conductance compared to when the fake hand was hammered before the tactile stimulation^20^. Thus, the skin conductance response (SCR) to a threatening stimulus is considered a physiological measure of illusory body ownership. In line with this, we adapted a method to assess ownership illusions for the two virtual hands in our study by virtually stabbing the hands of the joint avatar at the end of each session and recording the levels of skin conductance of the participants in response. The participants were instructed to reach the targets and keep the hand touching the target until it erases before returning their hands back to the original position. In the last target reach at the end of each session, we displayed a stimulus of a knife stabbing one of the hands of the joint avatar to measure startle responses using the skin conductance response (Fig. 3). We hypothesized that the change in skin conductance would be higher when the controlled arm is stabbed with the virtual knife compared to when the non-controlled arm is stabbed (H3). We also hypothesized there to be a difference in skin conductance levels towards the non-controlled arm between the three goal conditions (H4).

### H3

SCR is higher when the controlled arm is threatened than when the non-controlled arm is threatened.

### H4

SCR when the partner-controlled arm is threatened is higher while sharing the same goal with the partner compared to having separate goals and in the case of having separate goals, SCR when the partner-controlled arm is threatened is higher when the partner’s goal is visible compared to when it is invisible.

Participants answered a questionnaire regarding senses of body ownership and agency at the end of each session.

## Results

### Senses of body ownership and agency towards the non-controlled arm were higher in same goal and different goals: visible conditions compared to the different goals: invisible condition

**Figure 4:**
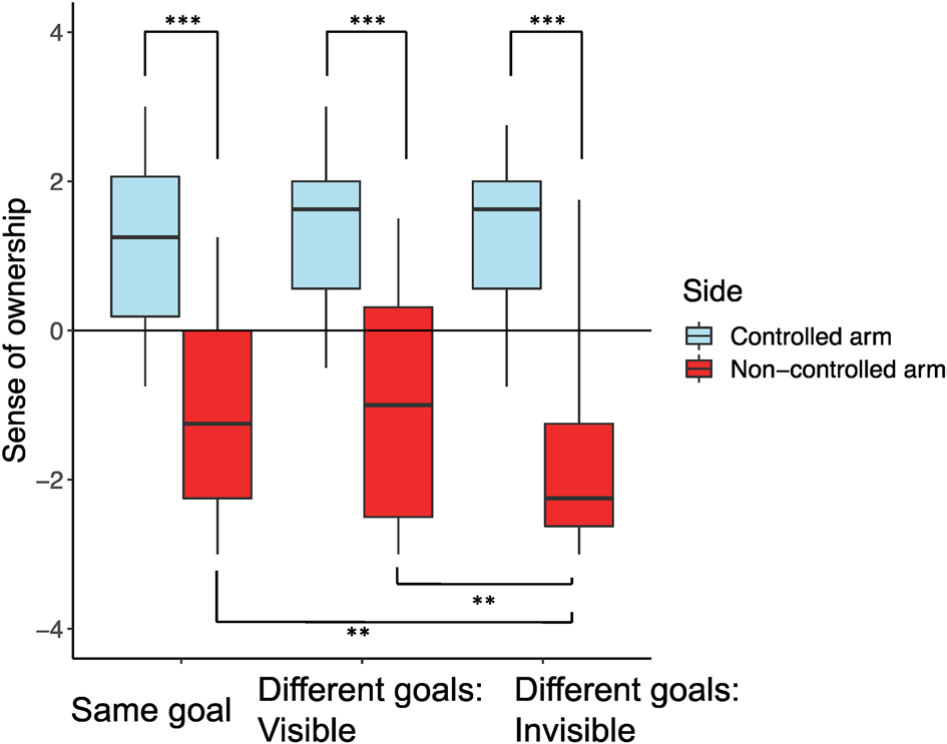
Sense of body ownership towards the controlled and non-controlled arms of the joint avatar in same goal, different goals: visible and different goals: invisible conditions. The ownership towards the non-controlled arm in the same goal and different goals: visible conditions were significantly higher than the ownership towards the non-controlled arm in the Different goals: invisible condition. In all three conditions, the ownership scores towards the controlled arm were significantly higher than the ownership scores towards the non-controlled arm.

**Figure 5:**
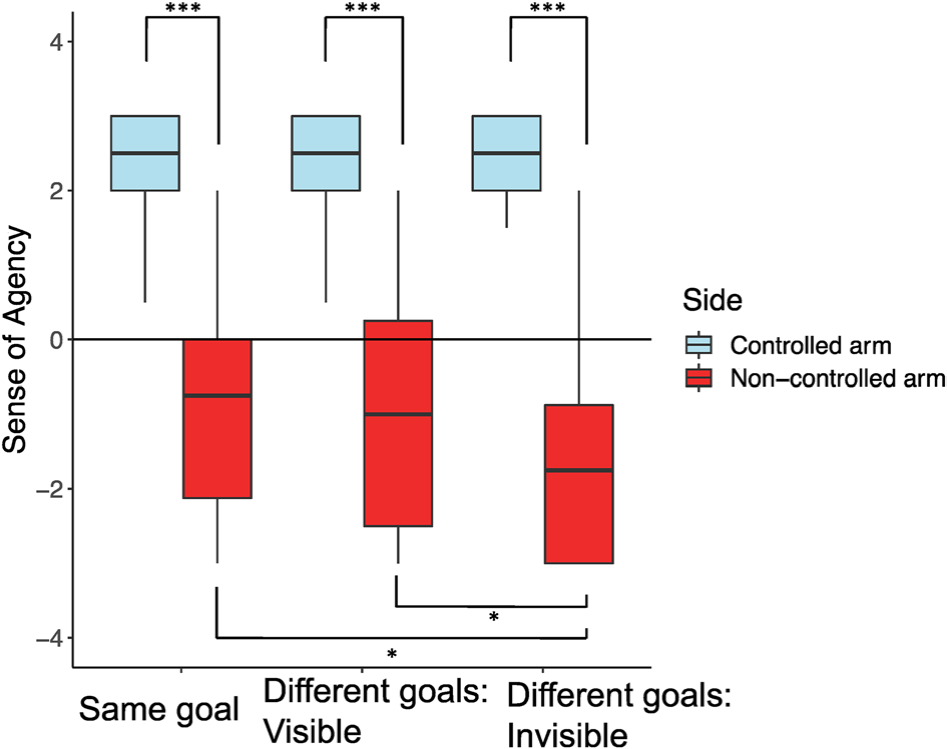
Sense of agency towards the controlled and non-controlled arms of the joint avatar in same goal, different goals: visible and different goals: invisible conditions. The agency towards the non-controlled arm in the same goal and different goals: visible conditions were significantly higher than the agency towards the non-controlled arm in the different goals: invisible condition. In all three conditions, the agency scores towards the controlled arm were significantly higher than the agency scores towards the non-controlled arm.

The data were categorized as “controlled arm” (the arm the participant controlled) and “non-controlled arm” (the arm that was controlled by the partner of the participant). For example, the ownership score towards the right arm of the joint avatar given by the participant who controlled the right arm of the joint avatar was categorized as the ownership for the “controlled arm” while his ownership score for the left arm of the joint avatar was categorized as ownership for the “non-controlled arm” (and vice versa for the participant who controlled the left arm of the joint avatar). Figures 4 and 5 summarize the results of the questionnaires.

We calculated the sense of body ownership (Fig. 4) towards the two arms of the joint avatar in the three conditions (Same goal, Different goals: Visible, Different goals: Invisible). For the questionnaire scores of both body ownership and agency, we first tested if the data violated normality, and then applied non-parametric ANOVA (2 controlled/non-controlled arms × 3 goal conditions) if the data deviated from normality. All simple main effects were tested if the interaction was significant, and the post-hoc multiple comparisons were performed among three goal conditions if the simple main effect was significant. These procedures of analysis were determined before conducting the experiment.

A two-way repeated measures ANOVA with ART (aligned rank transformation) procedure^21^ was conducted since some of the data significantly deviated from normality according to the results of Shapiro–Wilk tests (controlled arm for Same goal: W = 0.958, *p* = 0.500, non-controlled arm for Same goal: W = 0.933, *p* = 0.180, controlled arm for Different goals: Visible: W = 0.874, *p* = 0.014, non-controlled arm for Different goals: Visible: W = 0.914, *p* = 0.076, controlled arm for Different goals: Invisible: W = 0.911, *p* = 0.067, non-controlled arm for Different goals: Invisible: W = 0.844, *p* = 0.004). There was a significant interaction between the condition and arm (F(2,38) = 7.622, *p* = 0.002, $\eta_{p}^{2}$ = 0.286).

Significant simple main effects on the side condition were obtained in all goal conditions, and the sense of ownership was higher in the controlled arm condition than the non-controlled arm condition (the Same goal: F(1,19) = 78.641, *p* < 0.001, $\eta_{p}^{2}$ = 0.805, the Different goals: Visible: F(1,19) = 96.404, *p* < 0.001, $\eta_{p}^{2}$ = 0.835, the Different goals: Invisible: F(1,19) = 113.49, *p* < 0.001, $\eta_{p}^{2}$ = 0.857).

Significant simple main effects on the goal condition were seen only in the non-controlled arm (F(2,38) = 9.292, *p* < 0.001, $\eta_{p}^{2}$ = 0.328). Tukey post hoc tests confirmed that ownership was significantly higher in the Same goal condition compared to Different goals: Invisible condition (*p* = 0.0015, d = 1.201), and significantly higher in the Different goals: Visible condition compared to Different goals: Invisible condition (*p* = 0.0021, d = 1.159).

We calculated the sense of agency (Fig. 5) towards the two arms of the joint avatar in the three conditions (Same goal, Different goals: Visible, Different goals: Invisible). A two-way repeated measures ANOVA with ART (aligned rank transformation) procedure^21^ was conducted since some of the data significantly deviated from normality according to the results of Shapiro–Wilk tests (controlled arm for Same goal: W = 0.827, *p* = 0.002, non-controlled arm for Same goal: W = 0.935, *p* = 0.194, controlled arm for Different goals: Visible: W = 0.755, *p* < 0.001, non-controlled arm for Different goals: Visible: W = 0.907, *p* = 0.055, controlled arm for Different goals: Invisible: W = 0.815, *p* = 0.001, non-controlled arm for Different goals: Invisible: W = 0.872, *p* = 0.013). There was a significant interaction between the condition and arm (F(2,38) = 8.141, *p* = 0.001, $\eta_{p}^{2}$ = 0.300).

Significant simple main effects on the side condition were obtained in all goal conditions, and the sense of agency was higher in the controlled arm condition than the non-controlled arm condition (the Same goal: F(1,19) = 94.509, *p* < 0.001, $\eta_{p}^{2}$ = 0.833, the Different goals: Visible: F(1,19) = 157.38, *p* < 0.001, $\eta_{p}^{2}$ = 0.892, the Different goals: Invisible: F(1,19) = 174.04, *p* < 0.001, $\eta_{p}^{2}$ = 0.902).

Significant simple main effects on the goal condition were seen only in the non-controlled arm (F(2,38) = 0.211, *p* = 0.011, $\eta_{p}^{2}$ = 0.211). Tukey post hoc tests confirmed that agency was significantly higher in the Same goal condition compared to Different goals: Invisible condition (*p* = 0.0181, d = 0.906), and significantly higher in the Different goals: Visible condition compared to Different goals: Invisible condition (*p* = 0.0308, d = 0.837).

### Skin conductance responses were higher towards the controlled arm than towards the non-controlled arm, but not different among goal conditions

**Figure 6:**
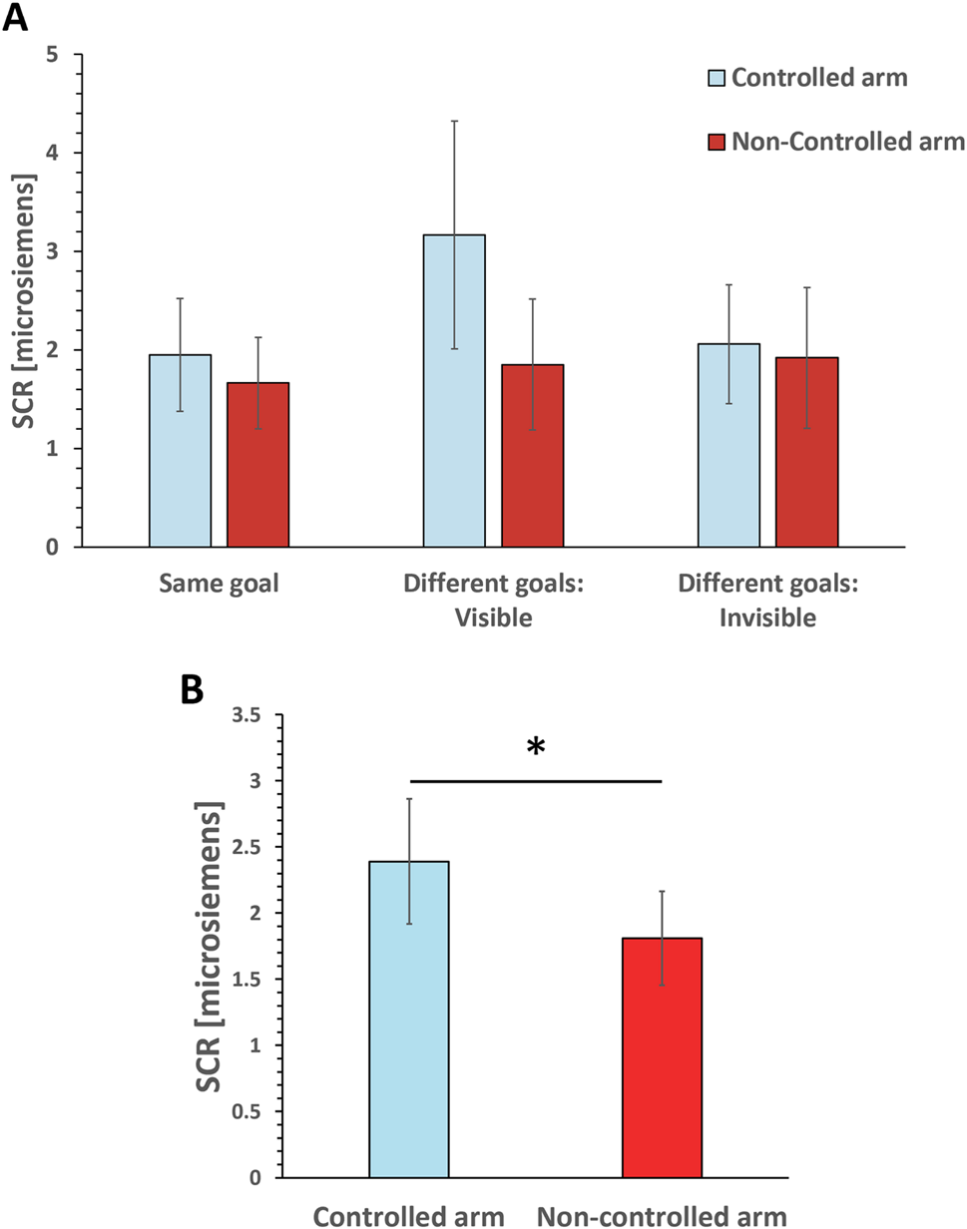
Skin conductance response. (**A**) Skin conductance response when the knife stabbed controlled or non-controlled arm at the end of each condition. (**B**) The three goal conditions were merged because neither the main effect of goal condition nor the interaction was significant. The skin conductance response was higher when the knife stabbed the controlled arm compared to when it stabbed the non-controlled arm.

Skin conductance response of each participant was calculated every time the threatening stimulus (virtual knife) appeared, as the difference between the maximum value detected in a 10 s post-stimulus time window and the baseline calculated as the average value of a 5 s pre-stimulus time window at a sample rate of 1000 Hz. We assumed the SCR data as parametric data and applied the parametric ANOVA. We firstly tested the sphericity of the data, and if there was a lack of sphericity, the reported values were adjusted using the Greenhouse–Geisser correction. Two-way repeated measures ANOVA showed no significant interaction between the goal condition and the arm. A significant main effect in the arm showed that the skin conductance response towards the controlled arm was significantly higher than that towards the non-controlled arm (F(1,19) = 6.8201, *p* = 0.017, $\eta_{p}^{2}$ = 0.264). However, no main effect of the goal condition or the interaction was observed. Figure 6A shows the calculated average skin conductance values when the controlled/non-controlled arm of the joint avatar was stabbed with the virtual knife; the three goal conditions were merged (Fig. 6B) because neither the main effect of goal condition nor the interaction was significant.

### Exploratory analyses including effects of dyads

The experiment was performed by 10 dyads. Participants were assigned randomly to right side or left side of the joint avatar. Thus, we performed additional statistical tests including the effect of dyads as exploratory analyses.

For the sense of body ownership, a three-way repeated measures ANOVA with ART was performed with 2 controlled/non-controlled arm conditions, 3 goal conditions (same goal, different goals: visible, different goals: invisible), and 2 dyad roles (right/left) since the data deviated from normality (Shapiro–Wilk tests, *p* < 0.05). The sample size was 10 dyads. We found the main effect of the arm condition (F(1,9) = 405.76, *p* < 0.001, $\eta_{p}^{2}$ = 0.978) and a significant interaction between the condition and arm (F(2,81) = 5.876, *p* = 0.004, $\eta_{p}^{2}$ = 0.127). There was another interaction between the arm and dyad role (F(1,81) = 6.894, *p* = 0.010, $\eta_{p}^{2}$ = 0.078). Significant simple main effects on the arm at goal condition were obtained in all goal conditions, and the sense of ownership was higher in the controlled arm condition than the non-controlled arm (the Same goal: F(1,29) = 30.421, *p* < 0.001, $\eta_{p}^{2}$ = 0.512, the Different goals: Visible: F(1,29) = 31.465, *p* < 0.001, $\eta_{p}^{2}$ = 0.520, the Different goals: Invisible: F(1,29) = 62.676, *p* < 0.001, $\eta_{p}^{2}$ = 0.684). Significant simple main effects on the goal condition were seen neither in the controlled arm (F(2,48) = 0.143, *p* = 0.867, $\eta_{p}^{2}$ = 0.006) nor the non-controlled arm conditions (F(2,48) = 2.292, *p* = 0.112, $\eta_{p}^{2}$ = 0.005). A significant simple main effect on the dyad role was observed on the controlled arm condition where participants who controlled the left arm of the joint avatar showed a significantly higher ownership towards the controlled arm compared to participants who controlled the right arm (F(1,49) = 23.234, *p* < 0.001, $\eta_{p}^{2}$ = 0.322).

For the sense of agency, a three-way repeated measures ANOVA with ART was performed with 2 control/non-control arm conditions, 3 goal conditions (same goal, different goals: visible, different goals: invisible), and 2 dyad roles (right/left) since the data deviated from normality (Shapiro–Wilk tests, *p* < 0.05). We found the main effect of the arm condition (F(1,9) = 322.64, *p* < 0.001, $\eta_{p}^{2}$ = 0.973). We found a nearly significant interaction between the condition and arm (F(2,81) = 3.001, *p* = 0.055, $\eta_{p}^{2}$ = 0.069). However, no significant simple main effects were observed. There was a significant main effect on arm meaning sense of agency towards the controlled arm was higher compared to the sense of agency towards the non-controlled arm.

For the SCR data, a three-way repeated measures ANOVA was performed with 2 control/non-control arm conditions, 3 goal conditions (same goal, different goals: visible, different goals: invisible), and 2 dyad roles (right/left). We found no significant interaction between the goal condition and the arm. A significant main effect in the arm showed that the skin conductance response towards the controlled arm was significantly higher than that towards the non-controlled arm (F(1,9) = 7.86, *p* = 0.021, $\eta_{p}^{2}$ = 0.466). There were no main effects of the dyad or the goal condition, or the interactions. The results were consistent with the original analysis.

## Discussion

We investigated whether sharing a goal or being able to see the partner’s goal affects the senses of body ownership and agency towards an arm controlled by a partner when two individuals perform three types of reaching tasks together using a joint avatar in VR, of which one individual fully controls the movements of the left arm while the other fully controls the movements of the right arm. Our main interest was on how the visibility and sharing of the partner’s goal affects the embodiment towards the arm controlled by the partner. We found that the sense of ownership and the sense of agency towards the partner-controlled arm were significantly higher when the goal was common or when the partner’s target was different and visible compared to when the partner’s goal was different and invisible to the participants.

The hypothesis H1 has been partly supported by the findings since the sense of ownership and the sense of agency towards the non-controlled arm were significantly higher in the common goal condition compared to the invisible different goals condition but were not higher compared to the visible different goals condition. The hypothesis H2 has been supported since the sense of ownership and the sense of agency towards the non-controlled arm were significantly higher in the visible different goals condition compared to the invisible different goals condition. Taken together, we conclude that whether the partner’s goal is visible or not is critical to increase the virtual embodiment towards the limb controlled by the partner.

According to Wegner’s theory of apparent mental causation^22^, “sense of agency” which is a main sub-component of sense of embodiment, arises if (1) an intention precedes an observed action (priority), (2) the intention is compatible with this action (consistency), and (3) the intention is the most likely cause of this action (exclusivity). Although these three conditions apply when the intention and the action belong to the same person, would sense of agency arise if a partner moves an arm of a person’s avatar to match with the intention of the person? Results from our experiment provide an answer to this question. During sharing one goal (same goal condition) as well as knowing the position of the partner’s goal while having two separate goals (different goals: visible condition), the agency towards the arm controlled by the partner (non-controlled arm) was significantly increased compared to the control condition in which the partner’s target was invisible while having two separate goals (different goals: invisible condition). This shows that sense of agency does indeed increase when a partner moves an arm of a person’s avatar to match with the intention of the person. Interestingly, in addition to sense of agency, ownership towards the partner’s arm was also significantly increased in a similar manner between the three conditions. Overall, these results suggest that being able to see the partner’s target increases sense of embodiment towards the arm controlled by the partner satisfying the hypothesis H2 described in the introduction.

However, among same goal and different goals: visible conditions, there were no significant differences in ownership or agency towards the non-controlled arm. This suggests that being able to know the partner’s objective increases embodiment towards the arm controlled by the partner regardless of the shared or divided nature of the task (equally increased in both cases compared to the control condition). The previously mentioned (in “Introduction” section) study in the real environment has shown that hearing instructions followed by consistent action increases the feeling of control towards another’s hand (vicarious agency)^12^. In our experiment, allowing the participants to see the partner’s target (while sharing one goal or having two separate goals) provides instructions visually regarding the action about to be performed by the partner-controlled hand and the results showed that it does indeed increase the feeling of control (sense of agency). Thus, our results are basically consistent with the vicarious-agency study while introducing a visual method of giving instructions instead of language/auditory instructions. However, it is an open question whether the language instruction and the visual target have the same level of effects or whether they are based on the same mechanisms. This issue should be further investigated in a future study.

Furthermore, the senses of agency and ownership were both significantly higher towards the controlled arm than towards the non-controlled arm in all three conditions. Supporting these questionnaire results physiologically, skin conductance levels were also significantly higher when the controlled arm was virtually stabbed with a knife compared to when the virtual non-controlled arm was stabbed in all three conditions. Thus, the hypothesis H3 is supported by these results. However, between the three conditions there was no significant difference among the skin conductance levels towards either of the arms of the joint avatar as we hypothesized. Therefore, hypothesis H4 is not supported.

In general, these results show that embodiment towards an arm fully controlled by a partner was still significantly lower compared to a fully controlled arm even with goal sharing or knowing the partner’s intentions behind arm movements. Therefore, while we conclude visibility of the partner’s target (which conveys the partner’s intention for arm movements) to be a factor affecting embodiment, we also do point out that visibility alone is not sufficient to induce a level of ownership or agency to an equal level felt towards a fully controlled arm. To the best of our knowledge, there is no comparable study investigating senses of ownership and agency towards an arm controlled by another to evaluate the effect of knowing the partner’s intentions. However, the asynchronous condition in González-Franco et al.^6^ is similar to our non-controlled condition due to the use of pre-recorded motion which simulates a closer movement to a partner-controlled limb. They reported the scores 1.5 and 2.3 (depending on order of conditions) for body ownership and, 1.2 and 2.2 for sense of agency on the scale from 1 (disagree strongly) to 5 (agree strongly). The transformed values of their results to our 7-point Likert scale are − 2.25 and − 1.05 for body ownership and − 2.7 and − 1.2 for sense of agency. Our median values for the body ownership were − 1.25 (same goal), − 1.0 (visible different goal), and − 2.25 (visible different goal), and data for the sense of agency were − 0.75 (same goal),  − 1.0 (visible different goal), and − 1.75 (visible different goal). It is difficult to compare these results due to differences of purpose and methods, but our result of the same goal and the visible different goal conditions might be slightly higher than the previous study.

Even though the embodiment towards partner-controlled arms is low in general, coupled with other factors affecting embodiment towards such limbs, it may be possible to increase embodiment further. In the present study, we utilized the shared goal or intention based on the joint action studies^13^ and the vicarious agency study^15,16^, but assuming by the low ownership and agency scores towards the partner-controlled arm, the dyads in the current joint body avatar may not have felt a strong “we-mode”^17^ feeling overall. Therefore, future studies investigating other factors affecting embodiment towards limbs such as personality traits/behavior of the partner, different kinds of haptic feedbacks towards the limbs or body, level of trust between the dyads etc. must be considered. Investigating such factors in isolation as well as coupled together in various combinations may provide further insights on enhancing we-mode and sense of embodiment towards limbs controlled by others.

One may argue that the participants were not independent since they were organized in dyads where the partner’s behavior may have had an effect on levels of embodiment. Thus, we exploratorily analyzed the data by adding the dyad role (the role of controlling either the left or the right arm of the avatar) as an independent variable and analyzed the results as dyads. The results were similar to the original analyses, but some results were different. For the body ownership, the interaction between the arm and dyad role was significant, but the simple main effects on the goal condition were significant neither in the controlled arm nor the non-controlled arm conditions. This may be due to the small sample size of dyads (N = 10). The left-am participants felt higher body ownership than the right-arm participants only in the controlled arm condition. This may be caused by the right hemisphere (and the left hand) dominance of the sense of body ownership^23^. For the sense of agency, the interaction between arm and goal conditions did not show significance. This may also be due to the small sample size of dyads. Our experimental plan including the sample size was based on similar studies of virtual co-embodiment where experiments were performed by participant dyads^7,8^. Therefore, while the main analysis of this study assumes each participant to be independent, we need to consider the effect of dyads or dependency of embodiment of participants on their partners in future studies regarding co-embodiment.

According to some previous research, avatar tracking error^24^ and motion artifacts^25^ have been known to decrease user experience and sense of agency toward a self-avatar. The action-effect delay is widely used to disrupt the sense of agency^26^. It is an interesting question whether the sense of agency can be improved if we consider the tracking errors, motion artifacts, or delay as system’s or computer’s intention and know them visually or with other modalities. More generally, it should be investigated whether the shared intention for increasing sense of agency in the joint avatar must originate from human partners or not in the future.

Next, we would like to discuss about some future possibilities/applications of virtual co-embodiment with regards to the future of VR. Future cyberspaces and virtual societies such as the “metaverse” are expected to weaken the concepts of race, gender, and physical disability^27^. Virtual embodiment enables us to feel an illusory body ownership towards avatars in different skin color^28^, gender^29^, and size^30^, bodies that are invisible or transparent^31–35^, and non-human agents^36^ and animals^37^. We are interested in the idea of minimizing physical disabilities of users in the virtual worlds through specially designed virtual avatars^38^. Human movement augmentation such as supernumerary limbs, body-parts remapping, and exoskeletons expands physical abilities both for impaired and unimpaired individuals^39–42^. Virtual co-embodiments like the shared body^7,8^ and our joint body could expand human collaborations as a co-embodied avatar by combining different persons’ strengths and compensating weaknesses. In social VR platforms, with appropriate software developments and accurate body movement tracking, it would be possible for multiple users to embody one avatar and engage in various tasks together. This may allow disabled VR users to get support from others and increase work efficiency as well as user satisfaction in the future. Our results suggest that a person’s goal or intention of the co-embodiment avatar must be conveyed to the partner in vision or other modalities to maintain the least sense of agency to the other’s controlling limb.

Finally, we would like to mention some possible real-world applications of our results outside VR. Previous work has shown that upper limb amputees can be made to experience a rubber hand as a part of their own body by applying synchronous touches to the stump, which was out of view, and to the index finger of a rubber hand placed in full view^19^. Our findings may contribute to similarly inducing illusory senses of ownership and agency towards robotic or autonomous prosthetics of amputees through visual stimulation manipulation (possibly with AR glasses paired with autonomous prosthetics). For example, if the autonomous prosthesis system shows its target to be reached or grasped to the amputee user immediately before the action is performed, the user may feel a higher prosthesis embodiment which may improve usability and naturalness of such robotic/autonomous prosthetics.

## Limitations of the study

The findings regarding the embodiment towards the non-controlled arm among the three main experiment conditions were not supported by SCR results in this study. Therefore, further investigation of other physiological measures such as proprioceptive drifts and changes in body schema/body image due to experiencing of the joint avatar, may be more appropriate for assessing embodiment in future work.

The participant population was not very diverse with regards to gender and age in this study where all participants were male university students (90% of them were Japanese). A replication of this study with a population of more demographic, gender and age diversity may provide more universal and unbiased results^43^.

An Asian male avatar was used in the experiment because most of the participants were Asian (Japanese) males. Appearance similarity between the participants and the virtual avatar affects embodiments; cartoon-like virtual avatar mimicking the outfit of the participant is better than the photorealistic avatar or dissimilar cartoon-like avatar^44^. Thus, the similarity between the joint avatar and the individuals may affect the results. This issue should be further investigated in the future.

We tested the data normality with Shapiro–Wilk tests followed by parametric or non-parametric tests based on the normality tests before conducting the experiment. One may argue that it would be easier and more correct to check normality assumptions on the residuals of the ANOVA model.

## Methods

### Participants

Twenty university students as ten dyads (All males, mean age 22.3, SD 2.4, 19 right-handed) participated in the experiment as dyads. The sample size was determined by a power analysis with repeated measures ANOVA (6 within factors: 2 × 3 conditions), an effect size (f) of 0.25 (medium), an alpha of 0.05, and power of 0.80 using G*Power 3.1. The calculated sample size was 19, then we determined the sample size as 20 for making dyads. All participants were unaware of the hypothesis of the experiments and had normal or corrected-to-normal eyesight. They provided informed consent before the experiment. The methods of the experiment were approved by the Ethical Committee for Human-Subject Research at the Toyohashi University of Technology, and all methods were carried out in accordance with the relevant guidelines and regulations.

### Setup and apparatus

The movements of the two participants were measured by a motion capture system (Vicon Bonita10, 12 cameras, 1024 × 1027 pixels, 250 fps). Two computers with the same specifications received the processed information of the motion capture data and presented the virtual environment on head-mounted displays (HMD: HTC VIVE Pro Eye, 1440 × 1600 pixels per eye, 90 × 110 deg, 90 Hz refresh) of both participants. The virtual environment and experiment tasks were created using Unity (2017.4.1f1). BIOPAC MP 160 and wireless PPG and EDA amplifiers (BN-PPGED) were used to record the skin conductance response (SCR). A wireless transmitter (BN-PPGED) was placed on each participant’s wrist. Two disposable electrodes (EL507) were pasted to the distal phalanges of their middle and ring fingers. Two lead cables (BN-EDALEAD2) connected the wireless transmitter to the electrodes. The SCR data were recorded at 1000 Hz.

## Stimuli and conditions

**Figure 7:**
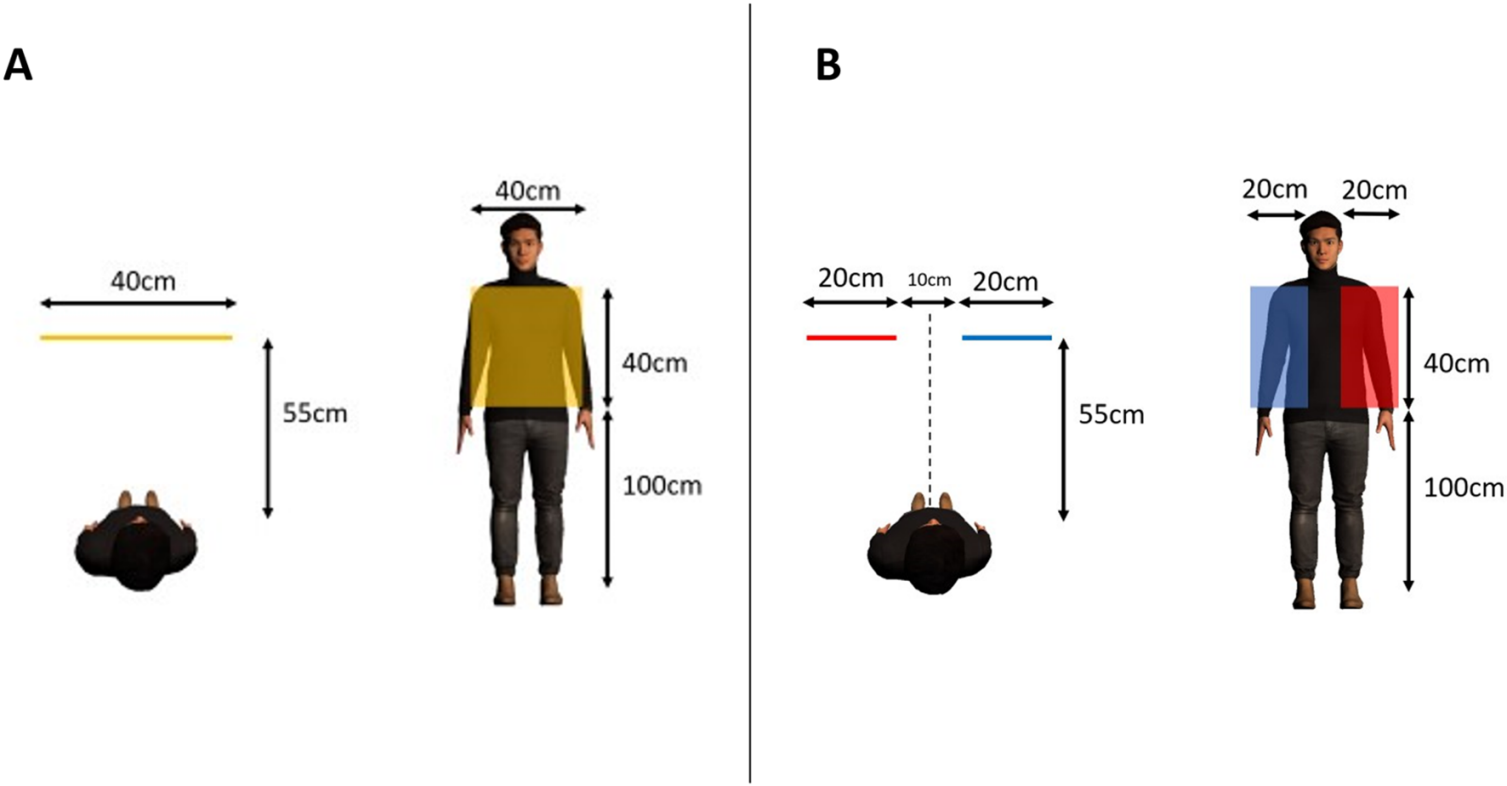
Target areas in each condition. (**A**) In the Same goal condition, yellow targets appeared at random positions inside the area shown in yellow. (**B**) In the different goals: visible and different goals: invisible conditions, the red targets that were to be reached with the left hand of the joint avatar appeared at random positions inside the area shown in red, and the blue targets that were to be reached with the right hand of the joint avatar appeared at random positions inside the area shown in blue.

The experiment consisted of three conditions. In each session, one participant controlled the left side of the joint avatar while the other controlled the right side throughout the experiment, both in first-person view. The virtual avatar used in this study was an Asian male avatar since 90% of the participants were Asian (Japanese) males. We named the three conditions “Same goal”, “Different goals: visible”, and “Different goals: invisible”. In the “same goal condition”, one yellow target appeared in front of the joint avatar and participants were asked to collaborate and touch the target with both hands of the joint avatar. In the different goals: visible condition, two targets (one blue and one red) appeared in front of the avatar and participants were asked to touch the blue target with the right hand and red target with the left hand of the joint avatar. Different goals: invisible condition was the same as different goals: visible condition, but the target of their partner was invisible for each participant. All targets were spheres of 5 cm diameter and were erased 200 ms after reached appropriately for each condition. After erasing, the next target appeared 2 s later. The targets appeared at random positions inside the areas shown in Fig. 7. Each session consisted of 100 reaches per participant and on the final reach of each session, a stimulus of a knife stabbing either the left or the right hand of the joint avatar was displayed.

## Procedure

The participants were instructed to reach the targets and keep the hand touching the target until it erases before returning their hands back to the original position. In the last target reach at the end of each session, we displayed a stimulus of a knife stabbing one of the hands of the joint avatar (see Fig. 3). To make the knife visible to both participants, the last target position was pre-chosen to be towards the center of the avatar in a clearly visible position and the final target/two targets was/were not erased on touch hence requiring the participants to keep touching the targets. We measured skin conductance response of participants during the stimulus of the knife to assess embodiment towards each arm of the avatar in each condition.

The experiment consisted of six sessions (3 conditions × 2 repetitions). Each condition was blocked as a session. In the two repetitions for each condition, left hand was stabbed at the end of one repetition while the right hand was stabbed in the other. The order of sessions was randomized for each dyad. One session lasted 5–7 min and after each session, both participants answered a questionnaire regarding how they felt about the left and right arms of the joint avatar. The questionnaire was based on a previous study (Table 1) ^45^. Questionnaire answers were accepted in a 7-point Likert scale from − 3 to + 3 where − 3 meant “Strongly disagree” and + 3 meant “Strongly agree”.

**Table 1:** Questionnaire items.

Q1	The movements of the virtual left arm seemed to be my movements
Q2	I felt as if the virtual left arm I saw was my own left arm
Q3	It seemed as if I felt the touch of the target sphere at the end of my left index finger
Q4	The movements of the virtual right arm seemed to be my movements
Q5	I felt as if the virtual right arm I saw was my own right arm
Q6	It seemed as if I felt the touch of the target sphere at the end of my right index finger

Using the answers, senses of ownership and agency towards left and right sides of the joint avatar for each condition were calculated as follows referring the Avatar embodiment standardized questionnaire by Peck, & Gonzalez-Franco ^45^.

$${\text{Sense}}\,{\text{of}}\,{\text{agency}}\,{\text{towards}}\,{\text{the}}\,{\text{left}}\,{\text{arm}}\, = \,{\text{Q1}}$$

$${\text{Sense}}\,{\text{of}}\,{\text{body}}\,{\text{ownership}}\,{\text{towards}}\,{\text{the}}\,{\text{left}}\,{\text{arm}}\, = \,\left( {{\text{Q2}} + {\text{Q3}}} \right)/{2}$$

$${\text{Sense}}\,{\text{of}}\,{\text{agency}}\,{\text{towards}}\,{\text{the}}\,{\text{right}}\,{\text{arm}}\, = \,{\text{Q4}}$$

$${\text{Sense}}\,{\text{of}}\,{\text{body}}\,{\text{ ownership}}\,{\text{towards}}\,{\text{the}}\,{\text{right}}\,{\text{arm}}\, = \,\left( {{\text{Q5}} + {\text{Q6}}} \right)/{2}$$

Skin conductance response was calculated as the difference between the maximum value detected in a 10 s post-stimulus (knife) time window and the baseline calculated as the average value of a 5 s pre-stimulus time window (for both participants in each session).

## Data Availability

All data generated or analyzed during this study are available at Mendeley Data. (http://dx.doi.org/10.17632/fnhvhb5b9z.1).

## References

[1] Kilteni K, Groten R, Slater M (2012). The sense of embodiment in virtual reality. Presence Teleoperators Virtual Environ..

[2] Botvinick M, Cohen J (1998). Rubber hands ‘feel’touch that eyes see. Nature.

[3] Gallagher S (2000). Philosophical conceptions of the self: Implications for cognitive science. Trends Cogn. Sci..

[4] Haggard P (2005). Conscious intention and motor cognition. Trends Cogn. Sci..

[5] Blanke O, Metzinger T (2009). Full-body illusions and minimal phenomenal selfhood. Trends Cogn. Sci..

[6] Gonzalez-Franco, M., Perez-Marcos, D., Spanlang, B., & Slater, M. (2010). The contribution of real-time mirror reflections of motor actions on virtual body ownership in an immersive virtual environment. In *2010 IEEE virtual reality conference (VR)* (pp. 111–114). IEEE.

[7] Fribourg R, Ogawa N, Hoyet L, Argelaguet F, Narumi T, Hirose M, Lécuyer A (2020). Virtual co-embodiment: Evaluation of the sense of agency while sharing the control of a virtual body among two individuals. IEEE Trans. Visual Comput. Graphics.

[8] Hagiwara T, Ganesh G, Sugimoto M, Inami M, Kitazaki M (2020). Individuals prioritize the reach straightness and hand jerk of a shared avatar over their own. iScience.

[9] Hagiwara, T., Katagiri, T., Yukawa, H., Ogura, I., Tanada, R., Nishimura, T., & Minamizawa, K. (2021). Collaborative Avatar Platform for Collective Human Expertise. *SIGGRAPH Asia 2021 Emerging Technologies*, 1–2.

[10] Hapuarachchi H., Ganesh, G., Hagiwara, T., and Kitazaki, M. (Under review). Connection force feedback in joint avatar increases embodiment towards a body part controlled by another.

[11] Hapuarachchi H, Kitazaki M (2022). Knowing the partner's objective increases embodiment towards a limb controlled by the partner, IEEE VR 2022 Conference, 12–16 March 2022, Poster. Proc. IEEE VR.

[12] Wegner DM, Sparrow B, Winerman L (2004). Vicarious agency: Experiencing control over the movements of others. J. Pers. Soc. Psychol..

[13] Sebanz N, Bekkering H, Knoblich G (2006). Joint action: Bodies and minds moving together. Trends Cogn. Sci..

[14] Vesper C, Abramova E, Bütepage J, Ciardo F, Crossey B, Effenberg A, Wahn B (2017). Joint action: Mental representations, shared information and general mechanisms for coordinating with others. Front. Psychol..

[15] Frischen A, Tipper SP (2004). Orienting attention via observed gaze shift evokes longer term inhibitory effects: Implications for social interactions, attention, and memory. J. Exp. Psychol. Gen..

[16] Tollefsen D (2005). Let’s pretend! Children and joint action. Philos. Soc. Sci..

[17] Gallotti M, Frith CD (2013). Social cognition in the we-mode. Trends Cogn. Sci..

[18] Armel KC, Ramachandran VS (2003). Projecting sensations to external objects: Evidence from skin conductance response. Proc. R Soc. London Ser. B Biol. Sci..

[19] Ehrsson HH, Rosén B, Stockselius A, Ragnö C, Köhler P, Lundborg G (2008). Upper limb amputees can be induced to experience a rubber hand as their own. Brain.

[20] Tsuji, T., Yamakawa, H., Yamashita, A., Takakusaki, K., Maeda, T., Kato, M., & Asama, H. (2013). Analysis of electromyography and skin conductance response during rubber hand illusion. In *2013 IEEE Workshop on Advanced Robotics and its Social Impacts* (pp. 88–93). IEEE.

[21] Wobbrock, J. O., Findlater, L., Gergle, D., & Higgins, J. J. (2011). The aligned rank transform for nonparametric factorial analyses using only anova procedures. In *Proceedings of the SIGCHI Conference on Human Factors in Computing Systems* (pp. 143–146).

[22] Wegner DM, Wheatley T (1999). Apparent mental causation: Sources of the experience of will. Am. Psychol..

[23] Ocklenburg S, Rüther N, Peterburs J, Pinnow M, Güntürkün O (2011). Laterality in the rubber hand illusion. Laterality.

[24] Toothman, N., & Neff, M. (2019). The impact of avatar tracking errors on user experience in VR. In 2019 IEEE Conference on Virtual Reality and 3D User Interfaces (VR) (pp. 756–766). IEEE.

[25] Koilias A, Mousas C, Anagnostopoulos CN (2019). The effects of motion artifacts on self-avatar agency. Informatics.

[26] Wen W (2019). Does delay in feedback diminish sense of agency? A review. Conscious. Cognit..

[27] Duan, H., Li, J., Fan, S., Lin, Z., Wu, X., & Cai, W. (2021). Metaverse for social good: A university campus prototype. In *Proceedings of the 29th ACM international conference on multimedia* (pp. 153–161).

[28] Peck TC, Seinfeld S, Aglioti SM, Slater M (2013). Putting yourself in the skin of a black avatar reduces implicit racial bias. Conscious. Cogn..

[29] Lopez, S., Yang, Y., Beltran, K., Kim, S. J., Cruz Hernandez, J., Simran, C., & Yuksel, B. F. (2019). Investigating implicit gender bias and embodiment of white males in virtual reality with full body visuomotor synchrony. In *Proceedings of the 2019 CHI Conference on human factors in computing systems* (pp. 1–12).

[30] Tambone R, Poggio G, Pyasik M, Burin D, Dal Monte O, Schintu S, Pia L (2021). Changing your body changes your eating attitudes: Embodiment of a slim virtual avatar induces avoidance of high-calorie food. Heliyon.

[31] Guterstam A, Gentile G, Ehrsson HH (2013). The invisible hand illusion: Multisensory integration leads to the embodiment of a discrete volume of empty space. J. Cogn. Neurosci..

[32] Guterstam A, Abdulkarim Z, Ehrsson HH (2015). Illusory ownership of an invisible body reduces autonomic and subjective social anxiety responses. Sci. Rep..

[33] Martini M, Kilteni K, Maselli A, Sanchez-Vives MV (2015). The body fades away: Investigating the effects of transparency of an embodied virtual body on pain threshold and body ownership. Sci. Rep..

[34] Kondo R, Sugimoto M, Minamizawa K, Hoshi T, Inami M, Kitazaki M (2018). Illusory body ownership of an invisible body interpolated between virtual hands and feet via visual-motor synchronicity. Sci. Rep..

[35] Kondo R, Tani Y, Sugimoto M, Inami M, Kitazaki M (2020). Scrambled body differentiates body part ownership from the full body illusion. Sci. Rep..

[36] Hoffmann, L., Bock, N., & vd Pütten, A. M. R. (2018). The peculiarities of robot embodiment (EmCorp-Scale): Development, validation and initial test of the embodiment and corporeality of artificial agents scale. In *2018 13th ACM/IEEE International conference on human-robot interaction (HRI)* (pp. 370–378). IEEE.

[37] Krekhov, A., Cmentowski, S., Emmerich, K., & Krüger, J. (2019). Beyond human: Animals as an escape from stereotype avatars in virtual reality games. In *Proceedings of the annual symposium on computer-human interaction in play* (pp. 439–451).

[38] Inami M, Uriu D, Kashino Z, Yoshida S, Saito H, Maekawa A, Kitazaki M (2022). Cyborgs human augmentation, cybernetics, and JIZAI body. Proc. Augment. Hum..

[39] Eden J, Bräcklein M, Ibáñez J, Barsakcioglu DY, Di Pino G, Farina D, Mehring C (2022). Principles of human movement augmentation and the challenges in making it a reality. Nat. Commun..

[40] Sasaki, T., Saraiji, M. Y., Fernando, C. L., Minamizawa, K. & Inami, M. Metalimbs: Multiple arms interaction metamorphism. In *ACM SIGGRAPH Emerging Technologies*, 1–2 (ACM, Los Angeles, California, USA, 2017).

[41] Umezawa K, Suzuki Y, Ganesh G, Miyawaki Y (2022). Bodily ownership of an independent supernumerary limb: An exploratory study. Sci. Rep..

[42] Kondo R, Tani Y, Sugimoto M, Minamizawa K, Inami M, Kitazaki M (2020). Re-association of body parts: Illusory ownership of a virtual arm associated with the contralateral real finger by visuo-motor synchrony. Front. Robot. AI.

[43] Peck TC, Sockol LE, Hancock SM (2020). Mind the gap: The underrepresentation of female participants and authors in virtual reality research. IEEE Trans. Vis. Comput. Graph..

[44] Jo, D., Kim, K., Welch, G. F., Jeon, W., Kim, Y., Kim, K. H., & Kim, G. J. (2017). The impact of avatar-owner visual similarity on body ownership in immersive virtual reality. In *Proceedings of the 23rd ACM Symposium on Virtual Reality Software and Technology* (pp. 1–2).

[45] Peck TC, Gonzalez-Franco M (2021). Avatar embodiment: A standardized questionnaire. Front. Virtual Real..

